# Reliability and Validity of Instruments for Assessing Perinatal Depression in African Settings: Systematic Review and Meta-Analysis

**DOI:** 10.1371/journal.pone.0082521

**Published:** 2013-12-10

**Authors:** Alexander C. Tsai, Jennifer A. Scott, Kristin J. Hung, Jennifer Q. Zhu, Lynn T. Matthews, Christina Psaros, Mark Tomlinson

**Affiliations:** 1 Center for Global Health, Massachusetts General Hospital, Boston, Massachusetts, United States of America; 2 Department of Psychiatry, Massachusetts General Hospital, Boston, Massachusetts, United States of America; 3 Harvard Medical School, Boston, Massachusetts, United States of America; 4 Department of Obstetrics and Gynecology, Beth Israel Deaconess Medical Center, Boston, Massachusetts, United States of America; 5 Division of Women’s Health, Brigham and Women’s Hospital, Boston, Massachusetts, United States of America; 6 Harvard Humanitarian Initiative, Cambridge, Massachusetts, United States of America; 7 Harvard College, Cambridge, Massachusetts, United States of America; 8 Division of Infectious Diseases, Massachusetts General Hospital, Boston, Massachusetts, United States of America; 9 Division of Infectious Diseases, Beth Israel Deaconess Medical Center, Boston, Massachusetts, United States of America; 10 Centre for Public Mental Health, Department of Psychology, Stellenbosch University, Stellenbosch, South Africa; University of Western Sydney, Australia

## Abstract

**Background:**

A major barrier to improving perinatal mental health in Africa is the lack of locally validated tools for identifying probable cases of perinatal depression or for measuring changes in depression symptom severity. We systematically reviewed the evidence on the reliability and validity of instruments to assess perinatal depression in African settings.

**Methods and Findings:**

Of 1,027 records identified through searching 7 electronic databases, we reviewed 126 full-text reports. We included 25 unique studies, which were disseminated in 26 journal articles and 1 doctoral dissertation. These enrolled 12,544 women living in nine different North and sub-Saharan African countries. Only three studies (12%) used instruments developed specifically for use in a given cultural setting. Most studies provided evidence of criterion-related validity (20 [80%]) or reliability (15 [60%]), while fewer studies provided evidence of construct validity, content validity, or internal structure. The Edinburgh postnatal depression scale (EPDS), assessed in 16 studies (64%), was the most frequently used instrument in our sample. Ten studies estimated the internal consistency of the EPDS (median estimated coefficient alpha, 0.84; interquartile range, 0.71-0.87). For the 14 studies that estimated sensitivity and specificity for the EPDS, we constructed 2 x 2 tables for each cut-off score. Using a bivariate random-effects model, we estimated a pooled sensitivity of 0.94 (95% confidence interval [CI], 0.68-0.99) and a pooled specificity of 0.77 (95% CI, 0.59-0.88) at a cut-off score of ≥9, with higher cut-off scores yielding greater specificity at the cost of lower sensitivity.

**Conclusions:**

The EPDS can reliably and validly measure perinatal depression symptom severity or screen for probable postnatal depression in African countries, but more validation studies on other instruments are needed. In addition, more qualitative research is needed to adequately characterize local understandings of perinatal depression-like syndromes in different African contexts.

## Introduction

Major depressive disorder is a major public health issue and accounts for a large proportion of the global burden of disease [1,2], especially among women of reproductive age [3]. When episodes occur during the antenatal or postnatal periods, maternal depression can compromise children’s physical health [4] and socio-emotional development [5]. These collateral impacts provide added impetus for alleviating the burden of perinatal depression in low- and middle-income countries [6,7]. However, the high burden of perinatal depression in many African countries [8,9] has not been matched by adequate mental health systems or human resources for mental health [10,11].

Global disparities in population mental health and mental health systems are paralleled by disparities in the evidence base supporting effective intervention. In a recent review of 11,501 trials to treat or prevent mental disorders, less than one percent of the studies was conducted in low-income countries [12]. To narrow the gap, more research is needed on interventions that can be delivered in non-hospital settings [13], such as stepped collaborative care [14,15]. The effectiveness and/or feasibility of implementing such care delivery models in low- and middle-income countries have only recently been established [16–20].

These new perinatal depression treatment and prevention strategies may require task shifting to non-specialist health workers and, therefore, more reliance on locally validated tools to support case identification or to measure changes in symptom severity. However, the typical arc of research in African settings consists of scale development in a Western setting, translation to the local language, back-translation to English in order to ensure accuracy of the translation, and then utilization without further assessment of the scale’s reliability and validity in the study’s context. It is not always clear that scale items can be literally translated and/or applied across cultures in such a straightforward fashion. To address these gaps in the literature, we performed a systematic review and meta-analysis of locally validated instruments used in African settings to screen for perinatal depression or to measure perinatal depression symptom severity. 

## Methods

### Ethics Statement

This study was reviewed by the Partners Human Research Committee and deemed exempt from full review because it was based on anonymous, public-use data with no identifiable information on participants.

### Study selection

The study protocol for this systematic review was not pre-registered. Our systematic evidence search, which was conducted January-May 2012, employed seven electronic databases: African Journals Online, the African Journal Archive, the Cumulative Index to Nursing and Allied Health Literature, Embase, the Medical Literature Analysis and Retrieval System Online (MEDLINE), PsycINFO, and the World Health Organization African Index Medicus. The specific search terms applied to these databases are listed in Table **S1**. In January 2013 we updated the MEDLINE search to identify articles published in the intervening 6-12 months. All citations were imported into the EndNote reference management software program (version X5, Thomson Reuters, New York, NY), and the “Find Duplicates” algorithm was used to identify duplicate references. Three study authors (ACT, JAS, JQZ) screened the titles and abstracts to identify potentially relevant articles for inclusion in the study. The full texts of these articles were examined for a final determination of relevance by the same three study authors. All disagreements were resolved by consensus. In addition, we searched the reference lists of articles selected for inclusion and queried colleagues in departments of psychiatry and psychology at other African academic institutions, in order to identify additional potentially relevant articles for inclusion.

To be included in this review, studies had to meet each of the following three criteria: (a) the study sample consisted of women living in African countries; (b) a questionnaire was used to screen study participants for major depressive disorder or to measure depression symptom severity, either during pregnancy or after delivery; and (c) the reliability and/or validity of the questionnaire was assessed. There were no language restrictions. Although the postnatal-onset specifier in the *Diagnostic and Statistical Manual of Mental Disorders* [21] describes a four-week onset, in practice this is generally considered to be arbitrary or overly restrictive [22]. Many research studies have permitted onsets of up to 12 months postnatally [23,24]. Therefore, for studies assessing depression after delivery, we accepted any author definition of postnatal-onset depression. 

A wide range of reliability and validity evidence was considered acceptable for inclusion. We categorized these into five broad domains:

1. Content validity: evaluations of scale content to ensure that scale items appropriately characterized a perinatal depression-like syndrome, e.g., through translation and/or adaptation of an instrument developed in another setting [25] or through qualitative research to develop a new instrument;
2. Reliability: analyses of the reproducibility of scale measurements, e.g., between raters (inter-rater reliability) or from one measurement to the next (test-retest reliability)
3. Internal structure: analyses of internal consistency to assess the extent to which scale items measure the same latent construct
4. Construct validity: confirming hypothesized relationships between the measurement scale and conceptually distinct constructs (convergent validity) or, alternatively, demonstrating the hypothesized lack of a relationship (discriminant validity)
5. Criterion-related validity: confirming hypothesized relationships between the measurement scale and “gold standard” reference criteria, either assessed simultaneously (concurrent validity) or at a subsequent time point (predictive validity)

### Data extraction and quality assessment

Two study authors (JAS, JQZ) independently abstracted data from non-overlapping subsets of the included reports, with all data reviewed by a third study author (ACT). Because the two data abstractors reviewed non-overlapping subsets of the included reports, no agreement statistics were calculated. For each report, data were extracted on the characteristics of the study population, including sampling strategy, sample size, inclusion criteria, instrument assessed, and type of reliability and/or validity evidence provided. For studies assessing criterion-related validity, data were extracted on the numbers of participants classified as true positives, true negatives, false positives, and false negatives, as well as items necessary to assess study quality according to the revised Quality Assessment of Diagnostic Accuracy Studies (QUADAS-2) [26]. Due to lack of variation in answers to several of the QUADAS-2 signaling questions, we limited quality assessment to three aspects of study design: whether the study avoided a case-control study design (i.e., in which the reference criterion is established in a subset of participants based on the results of the index test); whether the index test was administered in a uniform fashion; and whether the reference criterion was determined by an assessor who was blinded to the results of the index test.

### Statistical analysis

Due to substantial heterogeneity in the types of reliability and validity evidence provided, for most of the studies identified we did not attempt to make summary estimates using meta-analysis. As described in more detail below, we did, however, identify a critical mass of studies that provided evidence of criterion-related validity by comparing summary scores on the Edinburgh Postnatal Depression Scale (EPDS) [27] to “gold standard” reference criteria. For these studies, we constructed 2 x 2 tables for each cut-off score for which enough data were available and computed the sensitivity and specificity values. We then employed the bivariate random-effects model [28,29] to obtain pooled estimates of sensitivity and specificity and their associated 95% confidence intervals. At each cutoff score, we constructed summary receiver operating characteristic (ROC) curves to produce a 95% confidence ellipse within the ROC curve space [30]. Between-study heterogeneity was assessed with the *I* ^2^ statistic for the pooled diagnostic odds ratio [31]. To investigate excess heterogeneity, we used meta-regression to examine pooled sensitivity and specificity estimates stratified by three variables (which we selected *post hoc*): country, study setting, and timing of survey administration. We examined small sample size-related bias by plotting the logarithm of the diagnostic odds ratios against the inverse square root of the effective sample size and by fitting the accompanying regression model of the logarithm of the diagnostic odds ratios against the inverse square root of the effective sample size, weighting by the effective sample size [32]. All statistical analyses were implemented with the use of the Stata software package (version 12.1, StataCorp LP, College Station, Tex.).

## Results

**Figure 1:**
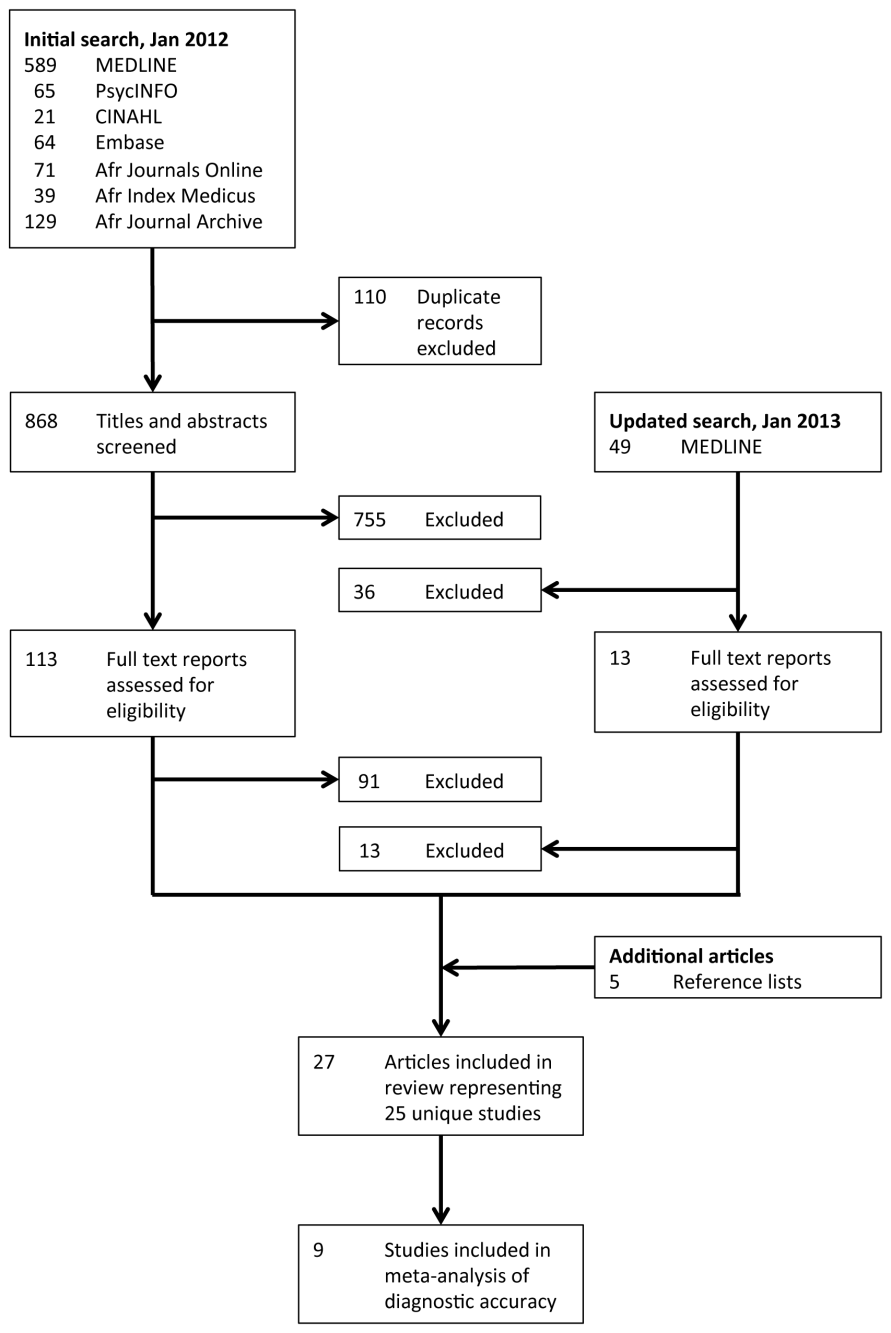
Quality of Reporting of Meta-Analyses (QUORUM) flow chart depicting the number of reports screened and included in the systematic review.

The initial set of search algorithms yielded a total of 978 records, of which 110 were duplicates (Figure **1**). After reviewing the remaining 868 records, we excluded 755 records on the basis of the title and abstract screening. We then retrieved 113 reports, including peer-reviewed journal articles and doctoral dissertations, for full text review. Of these, 90 reports were excluded because they did not provide evidence of reliability or validity of an instrument used to assess perinatal depression. One article appeared to be of relevance [33] but two attempts, spaced over four weeks, to obtain additional data from the study authors were not successful. In January 2013 the MEDLINE search was updated, yielding an additional five journal articles for inclusion. A total of 25 unique studies, reported in 26 journal articles and 1 doctoral dissertation, were included in this review (Table **S2**). 

Summary statistics for the sample are provided in Table **1**. The 25 studies enrolled 12,544 women living in nine different North African and sub-Saharan African countries, with Nigeria and South Africa accounting for more than one-half of the studies. The median sample size was 227 (interquartile range [IQR], 144-500). The predominant setting from which participants were recruited was the outpatient clinic or the community. In 10 studies (40%), the instruments were administered during pregnancy, while 15 studies (60%) assessed depression during the postnatal period. Among the latter, the largest percentage of studies assessed depression at 6-11 weeks postnatally (7/15 [47%]), two studies (13%) assessed depression at six months postnatally, and six studies (40%) did not specify a specific time window.

**Table 1:** Summary statistics (N=25 unique studies).

Study characteristic	Number (percent) or median (interquartile range)	
Country of origin		
Nigeria	9	(36)
South Africa	5	(20)
Tanzania	3	(12)
Other† ^¶^	8	(32)
Number of study participants	227	(144-500)
Population		
Antenatal	10	(40)
Postnatal	15	(60)
Study setting‡		
Outpatient	17	(68)
Community	10	(40)
Inpatient	2	(8)
Instrument assessed‡		
Edinburgh Postnatal Depression Scale	16	(64)
General Health Questionnaire	3	(12)
K6/K10	3	(12)
Hopkins Symptom Checklist	2	(8)
Other	10	(40)
Type of evidence provided‡		
Criterion-related validity	21	(84)
Reliability	15	(60)
Construct validity	9	(36)
Content validity	7	(28)
Internal structure	4	(16)

^†^ Includes Burkina Faso, Democratic Republic of Congo, Ethiopia (2 studies), Ghana, Morocco, Zimbabwe (2 studies)

^‡^ Percentages may not add up to 100, as categories are not mutually exclusive

^¶^ Includes a 14-item instrument designed to screen for *Malady ya Souci* (a locally identified syndrome) [51], Beck Depression Inventory [73], Dar-es-Salaam Symptom Questionnaire [52], Hospital Anxiety and Depression Scale [74], Maternity Blues Scale [75], Montgomery-Asberg Depression Rating Scale [76], Patient Health Questionnaire [77], Self-Reporting Questionnaire [78], Shona Symptom Questionnaire [49], and Zung Self-Rating Depression Scale [79].

Altogether, 14 different instruments were assessed in these studies (Table **1**). The EPDS, assessed in 16 studies (64%), was the most frequently used instrument in our sample. No other instrument was used consistently across settings. The most frequently studied alternatives to the EPDS -- the General Health Questionnaire [34], the K6/K10 [35], and the Hopkins Symptom Checklist for Depression (HSCL) [36] -- were not designed specifically to evaluate symptoms of depression during pregnancy or during the postnatal period. Only three studies (12%) used instruments developed specifically for use in a given cultural setting. Aspects of content and construct validity were explored in relatively few studies. Among the 21 studies assessing criterion-related validity, a substantial minority contained design elements that could lead to bias: 10 studies (48%) employed a case-control study design, 11 studies (52%) did not feature uniform administration of the index test, and in 7 studies (33%) the reference criterion was not determined by an assessor who was blinded to the results of the index test (Table **2**).

**Table 2:** Quality assessment of studies assessing the criterion-related validity of instruments to screen for perinatal depression.

Citation	Country	Index Test	Language	Reference Criterion	Period (Timing)	Case-Control^†^	Uniform Test^‡^	Blinding^¶^
Abiodun and colleagues [80]	Nigeria	GHQ-30	Yoruba	PSE	Antenatal	Yes	No	Yes
Abiodun [81]	Nigeria	GHQ-12, HADS	Yoruba	PSE	Antenatal	Yes	No	Yes
Abiodun [82]	Nigeria	EPDS	Yoruba	PSE	Postnatal (6 wk)	Yes	No	Yes
Aderibigbe and Gureje [83]	Nigeria	GHQ-28	Yoruba	PAS	Antenatal	Yes	No	Yes
Adewuya and colleagues [84]	Nigeria	EPDS, BDI	Yoruba	SCID	Postnatal (6 wk)	Yes	No	Yes
Adewuya [85]	Nigeria	EPDS	Yoruba	SADS	Postnatal (8 wk)	No	Unclear	Unclear
Adewuya and colleagues [86]	Nigeria	EPDS	Yoruba	MINI	Antenatal	Yes	No	Yes
Agoub and colleagues [87]	Morocco	EPDS	Arabic	MINI	Postnatal (6 mo)	No	No	No
Baggaley and colleagues [88]	Burkina Faso	K10/K6	West African French, Moore, Dioula	Clinical interview	Postnatal (6 mo)	No	Yes	Yes
Bass and colleagues [51]	Democratic Republic of Congo	HSCL, EPDS, *Malady ya Souci*	Lingala, French	Key informant	Postnatal (unclear)	Yes	Yes	Unclear
Chibanda and colleagues [89]	Zimbabwe	EPDS	Shona	Clinical interview	Postnatal (6 wk)	No	Yes	Yes
Hanlon and colleagues [90]	Ethiopia	SRQ	Amharic	CPRS	Postnatal (unclear)	No	Yes	Unclear
Kaaya and colleagues [91]	Tanzania	HSCL	Kiswahili	SCID	Antenatal	No	Yes	Yes
Lawrie and colleagues [92]	South Africa	EPDS	English	Clinical interview	Postnatal (6 wk)	No	No	Yes
Nhiwatiwa and colleagues [50]	Zimbabwe	SSQ	Shona	CISR	Antenatal*	Yes	Yes	Yes
Rochat [93]	South Africa	EPDS	Zulu	SCID	Antenatal	No	Yes	Unclear
Spies and colleagues [94]	South Africa	K10/K6	Afrikaans	SCID	Antenatal	No	Yes	Unclear
Taiwo and Olayinka [95]	Nigeria	EPDS	Hausa	Clinical interview	Postnatal (6 wk)	Yes	No	Yes
Tesfaye and colleagues [96]	Ethiopia	K10/K6	Amharic	Clinical interview	Postnatal (unclear)	No	Yes	Yes
Uwakwe and Okonkwo [97]	Nigeria	EPDS	Igbo	Clinical interview	Postnatal (unclear)	No	No	Unclear
Weobong and colleagues [98]	Ghana	EPDS, SRQ, PHQ	Twi	CPRS	Postnatal (5-11 wk)	Yes	Yes	Yes

BDI = Beck Depression Inventory; CISR = Revised Clinical Interview Schedule; CPRS = Comprehensive Psychopathological Rating Scale; EPDS = Edinburgh Postnatal Depression Scale; GHQ = General Health Questionnaire; MINI = Mini International Neuropsychiatric Interview; PAS = Psychiatric Assessment Schedule; PSE = Present State Examination; SADS = Schedule for Affective Disorders and Schizophrenia; SCID = Structured Clinical Interview for the Diagnostic and Statistical Manual of Mental Disorders; SRQ = Self-Reporting Questionnaire; SSQ = Shona Symptom Questionnaire

^†^ A case-control study design is one in which the reference criterion is established in a subset of participants based on the results of the index test.

^‡^ A non-uniform test may result when the index test is not administered in a uniform fashion, e.g., in study design in which literate participants may self-administer the index test and illiterate participants are administered the index test by a trained interviewer.

^¶^ The reference criterion is administered and/or assessed without knowledge of the index test results.

* The index test assessed antenatally was employed to predict the reference criterion assessed postnatally.

### The Edinburgh Postnatal Depression Scale

The EPDS was the only instrument for which each of the five types of reliability or validity evidence (i.e., the types catalogued in this review) was obtained in a single country, South Africa (Table **3**). Among the studies, most provided evidence of criterion-related validity (14 [88%]) or reliability (12 [75%]), with fewer studies providing evidence of construct validity (6 [38%]) or content validity (5 [31%]). Among the 10 studies that estimated the internal consistency of the EPDS, the median estimated coefficient alpha was 0.84 (IQR, 0.71-0.87). 

**Table 3:** Number of studies assessing reliability and validity of the Edinburgh Postnatal Depression Scale, by country*.

Country	Criterion-related validity	Reliability	Construct validity	Content validity	Internal structure
Burkina Faso					
Democratic Republic of Congo	1	1	1	1	
Ethiopia	2	2	1	2	
Ghana	1	1			
Morocco	1				
Nigeria	6	4	3		
South Africa	2	3	1	2	1
Tanzania					
Zimbabwe	1	1			

* Row and column totals may not add up to N=25, as the cells are not mutually exclusive

**Figure 2:**
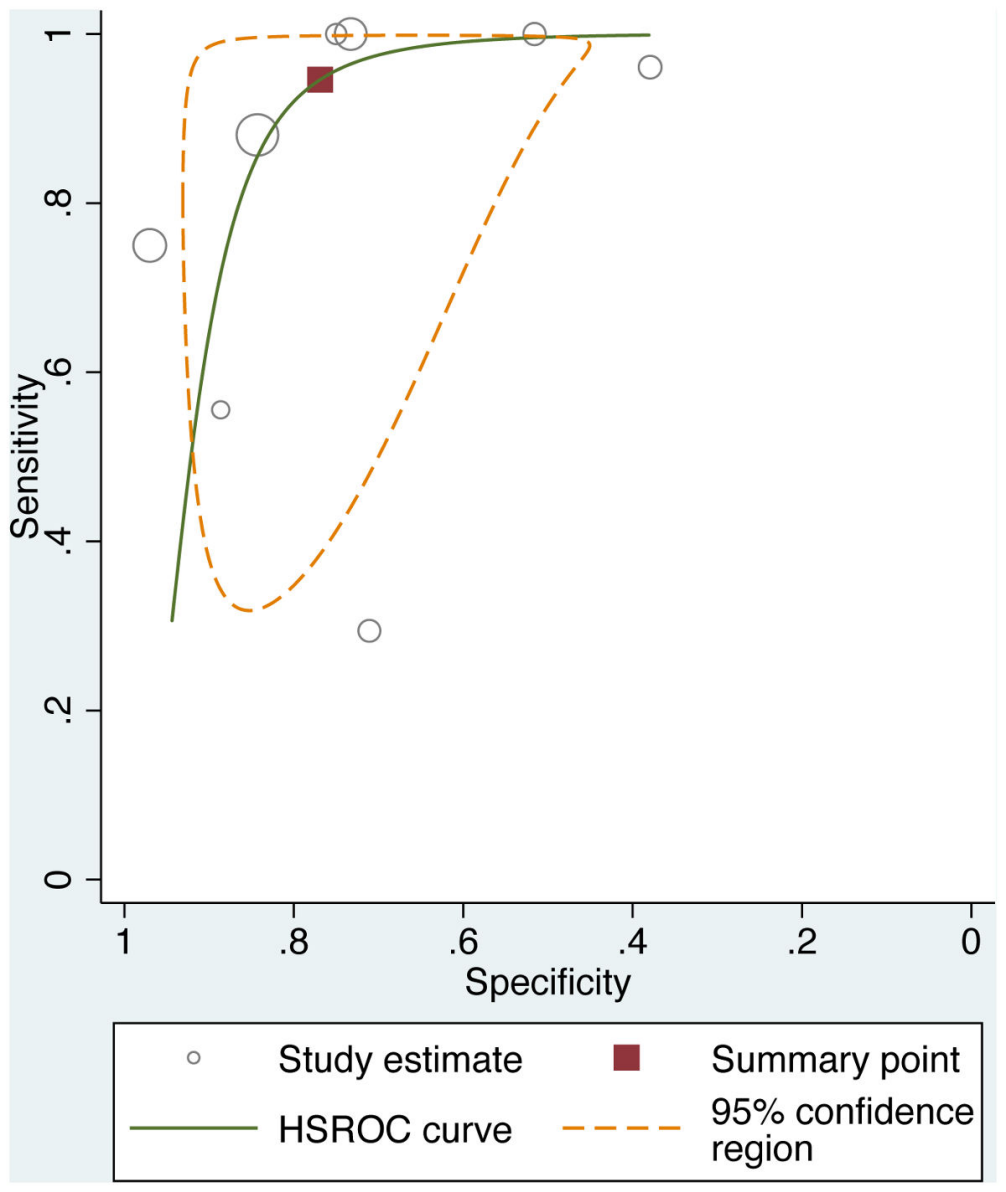
Summary ROC curve plot of diagnosis of perinatal depression based on EPDS ≥9. The solid line depicts the summary ROC curve from the bivariate random-effects model. The solid square depicts the summary operating point, i.e., summary values for sensitivity and specificity. The dotted line depicts the 95% confidence region for the summary operating point.

Among the 14 studies that provided evidence supporting criterion-related validity of the EPDS, two studies assessed antenatal depression and 12 studies assessed postnatal depression. When we summarized individual studies within ROC curve space for the commonly adopted cut-off score of ≥9, we observed that most studies gathered within an informative top left corner (Figure **2**). The summary ROC curves for three other cut-off scores, ≥7, ≥10, and ≥12, were visually similar (Figures S1, S2, and **S3**). These estimates suggested a pooled sensitivity of 0.94 (95% confidence interval [CI], 0.68-0.99) and a pooled specificity of 0.77 (95% CI, 0.59-0.88) at a cut-off score of ≥9 (Table **4**). In general, higher cut-off scores yielded greater specificity at the cost of lower sensitivity, with the exception of the cut-off of ≥7, at which both lower sensitivity and lower specificity were observed in comparison to those estimated at the cut-off of ≥9. There was substantial between-study heterogeneity, as suggested by *I* ^2^ values ranging from 85.5-95.4. The small sample of studies limited our ability to explore this heterogeneity, but across cut-off scores we found that studies conducted during the antenatal period had greater pooled sensitivity (P-values ranged from <0.01 to 0.73) and lower pooled specificity (P-values ranged from 0.03 to 0.16) compared to studies conducted during the postnatal period. Examination of log-diagnostic odds ratios plotted against inverse square root of effective sample size, and the accompanying regression tests, did not suggest small sample size-related bias (*P*-values ranged from 0.21 to 0.69) (Figures S4, S5, S6, and **S7**).

**Table 4:** Pooled estimates of sensitivity and specificity of the Edinburgh Postnatal Depression Scale, by cut-off score.

Cut-off score	Studies	Number of studies	Number of participants	Pooled sensitivity (95% CI)	Pooled specificity (95% CI)
≥7	Hanlon and colleagues [90], Lawrie and colleagues [92], Tesfaye and colleagues [96], Taiwo and Olayinka [95], Rochat [93]	5	701	0.89 (0.64-0.97)	0.51 (0.34-0.68)
≥9	Abiodun [82], Adewuya and colleagues [86], Chibanda and colleagues [89], Hanlon and colleagues [90], Lawrie and colleagues [92], Rochat [93], Taiwo and Olayinka [95], and Uwakwe and Okonkwo [97]	8	1,548	0.94 (0.68-0.99)	0.77 (0.59-0.88)
≥10	Abiodun [82], Adewuya and colleagues [86], Agoub and colleagues [87], Chibanda and colleagues [89], Hanlon and colleagues [90], Lawrie and colleagues [92], Rochat [93], Taiwo and Olayinka [95], and Weobong and colleagues [98]	9	1,627	0.84 (0.64-0.94)	0.81 (0.72-0.88)
≥12	Abiodun [82], Adewuya [85], Adewuya and colleagues [86], Agoub and colleagues [87], Chibanda and colleagues [89], Hanlon and colleagues [90], Lawrie and colleagues [92], Rochat [93], Taiwo and Olayinka [95], and Uwakwe and Okonkwo [97]	10	2,170	0.68 (0.47-0.83)	0.93 (0.87-0.97)

## Discussion

In this systematic review of instruments used to assess perinatal depression in African settings, we identified 25 unique studies of 14 different instruments. Most studies employed instruments developed in Western settings that were then applied to the African context, and few were newly created for a specific study context. Among the studies included in this review, we found that the EPDS was the most commonly evaluated instrument. The subset of our findings concerning the validity of the EPDS extends two previous systematic reviews focused solely on its sensitivity and specificity [37,38]: we undertook a more exhaustive search for African literature, we characterized a broader range of reliability and/or validity evidence, and we used the bivariate random-effects model to demonstrate the sensitivity-specificity tradeoff across a range of commonly adopted cut-off scores. Our findings have important implications for extending human resources for mental health in African settings.

Most of the instruments under investigation were originally developed using a sample recruited from a European or North American country, with the finalized instrument then translated for use in an African country. This approach can be described as a largely *etic* approach in which the construct of “depression” is promoted irrespective of culture, and has been criticized for assuming that the phenomenology of depression and Western categorizations of mental illness can be validly applied cross-culturally with minimum modification [39]. The use of a Western-derived instrument to assess perinatal depression in a different cultural context is not invalid, given that many symptoms of depression are universal. However, we also believe that *mental illness constructs* are not thought to be universal and are likely to be burdened with ethnocentric conceptualization. The experience of sadness or depressed mood may not even be a core presenting feature of affective disturbance in some cultural contexts [40,41]. Patel and colleagues [42], for example, reported the lack of conceptually equivalent terminology for describing depression among persons belonging to the Shona tribe, Zimbabwe’s largest indigenous group. Other qualitative studies have also shown substantial divergence between Western and local understandings of depression [43–45].

The *emic* approach to the study of depression, in contrast, emphasizes the evaluation of mental illness constructs from within a specific cultural context [46]. The field has long recognized the need for integrating both *etic* and *emic* validating criteria in a synthesis that investigates local explanatory models of mental illness while attempting to situate them within the dominant Western (biomedical) paradigm of classification [47]. Doing so has the potential to avoid culturally imposed assumptions about symptom meanings based on models of mental disorders derived from European and North American countries that can result in substantial errors in screening or measurement [48]. Patel and colleagues [49] adopted this hybrid approach to develop the 14-item Shona Symptom Questionnaire, which does not contain an item specifically addressing dysphoric mood even while its overall item composition shares many symptoms that are regarded as core features of the Western construct of depression (e.g., suicidal ideation, anhedonia). Therefore, we recommend that severity or case finding measures originally developed in Western settings be used with an emphasis on conceptual translation and adaptation with local idioms. 

We identified only three studies assessing the reliability and validity of perinatal depression instruments developed specifically for use in a given cultural setting, all of which employed qualitative methods to characterize conceptually valid local constructs and their psychosocial sequelae [50–52]. Local expressions were elaborated through in-depth interviews, worded as potential scale items, and added to item pools containing items derived from Western settings; psychometric analyses were then applied to the enriched item pools. Two of these newly derived instruments appeared to identify local depression-like syndromes [51,52], while the third was designed to detect general psychiatric morbidity [49]: between one-third and one-half of the scale items overlapped with items represented in standard instruments such as the HSCL, EPDS, or SRQ. The extent to which the use of this method generally yields instruments with *greater* reliability and/or validity is unclear. Only Bass and colleagues [51] compared their locally derived instrument to standard instruments such as the EPDS and HSCL. Their 14-item locally derived instrument had greater reliability compared to the EPDS and HSCL and had an area under the ROC curve value that was intermediate between those of the EPDS and HSCL, but the differences were not substantive in magnitude and no statistical significance testing was employed. This is an important gap in the literature that should be closed in subsequent studies. Nonetheless, given the attractive face validity of this method, we believe cross-cultural perinatal mental health research of this nature should be implemented more widely.

Notably, a large proportion of studies used generic depression instruments that were not specifically designed to measure symptoms of depression during pregnancy or during the postnatal period. We were unable to locate studies validating other frequently used scales for perinatal depression such as the Postpartum Depression Screening Scale [53] or the Bromley Postnatal Depression Scale [54]. To the extent there are potential phenotypic differences between perinatal vs. non-perinatal depression [55–57], the use of generic instruments may result in misclassification or measurement error. Certainly in some contexts a generic depression instrument may prove to be more sensitive and/or specific than a specific perinatal depression instrument. Whether specific or generic instruments have greater criterion-related validity is an empirical question, however, that could not be robustly answered by the data gathered in this review. 

Our systematic review points to an important gap in the literature that must be addressed in order to realize the programming implications of the findings from the identified research studies. Given the constrained mental health systems and human resources for mental health in many African settings [10,11], there is increasing recognition of the need to develop care delivery models that task-shift to non-specialist, lay health workers [19,58,59]. High-quality, randomized controlled trials conducted in sub-Saharan Africa suggest that the delivery of manualized psychosocial treatments by non-specialist, lay health workers is both feasible and effective [20,60,61], provided that cases can be identified and referred for treatment. However, community health workers’ workloads [62,63] may limit the extent to which they can effectively administer even short instruments to find cases or monitor responses to treatment. The use of *ultra-short* screening and measurement instruments (defined in one proposal as being limited to 4 items or fewer and requiring less than 2 minutes to administer [64]), perhaps facilitated with mobile technologies [65–67], may expedite a strategy of screening, treatment, and/or treatment response monitoring at scale. None of the studies identified in our review, however, provided evidence on the reliability or validity of such ultra-short instruments. Moreover, no studies have demonstrated that case-finding can be integrated into the routine course of lay health workers’ community-based outreach and wellness work. To avoid overwhelming mental health treatment programs with false positive referrals [68], more work is needed to establish the reliability and validity of ultra-short instruments.

### Limitations

Four limitations should be kept in mind when interpreting our findings. First, it is possible that our search protocol failed to uncover some studies, thereby leading us to underestimate the volume of medical and public health research aimed at validating perinatal depression scales in African settings. Second, and related to the above, our systematic review was not focused on anthropological research, e.g., we did not search AnthroSource or Anthropology Plus. Although the databases employed in our systematic evidence search included coverage of some social science journals and we identified qualitative studies published in medical and/or public health journals, the results of our systematic evidence search likely fail to represent the anthropological literature on this topic. Third, in the subsample of studies assessing criterion-related validity of the EPDS, we were unable to explain the large amount of between-study heterogeneity. Such a large degree of unexplained heterogeneity may lower our confidence in the findings from the meta-analysis. Fourth, even were we to assume construct validity as a given, a substantive proportion of studies assessing criterion-related validity contained design elements that could introduce bias. In general these methodological shortcomings might be expected to overstate the instruments’ diagnostic accuracy [69]. 

## Conclusions

In summary, we have identified 14 different instruments that have been developed or modified for assessing perinatal depression in specific African settings. Relatively more investigators have administered standard instruments while seeking to locally validate them, but such studies are still few in number. The EPDS was the focus of the largest number of studies, but our search protocol did not yield a sufficient number of other studies to permit robust conclusions about the comparative utility of different instruments. While these and other standard instruments could, with limitations [68,70–72], be employed to screen for perinatal depression in settings of elevated risk, the weak evidence base is a major barrier to sound programming for improving perinatal mental health in Africa. 

## Supporting Information

Checklist S1 **PRISMA checklist.** This checklist provides details in compliance with the Preferred Reporting Items for Systematic Reviews and Meta-Analyses (PRISMA) standard.
(DOC)

Figure S1 **Summary ROC curve plot.** The diagnosis of perinatal depression was based on the EPDS at the cut-off score ≥7, with pooled sensitivity and specificity calculated using bivariate meta-analysis.
(TIF)

Figure S2 **Summary ROC curve plot.** The diagnosis of perinatal depression was based on the EPDS at the cut-off score ≥10, with pooled sensitivity and specificity calculated using bivariate meta-analysis.
(TIF)

Figure S3 **Summary ROC curve plot.** The diagnosis of perinatal depression was based on the EPDS at the cut-off score ≥12, with pooled sensitivity and specificity calculated using bivariate meta-analysis.
(TIF)

Figure S4 **Asymmetry plot for EPDS at cut-off score ≥7.**
(TIF)

Figure S5 **Asymmetry plot for EPDS at cut-off score ≥9.**
(TIF)

Figure S6 **Asymmetry plot for EPDS at cut-off score ≥10.**
(TIF)

Figure S7 **Asymmetry plot for EPDS at cut-off score ≥12.**
(TIF)

Table S1 **Search terms applied to electronic databases.** All database searches were completed on January 27, 2012, with the exception of searches conducted using the African Journal Archive, African Journals Online, and the World Health Organization African Index Medicus (which were completed May 30, 2012). The Medical Literature Analysis and Retrieval System Online search was updated on January 23, 2013.
(PDF)

Table S2 **List of studies included in the review.**
(PDF)
