# Supplementary figures and images for: Reliability and Validity of Instruments for Assessing Perinatal Depression in African Settings: Systematic Review and Meta-Analysis
Source: PLoS One. 2013 Dec 10;8(12):e82521. doi: 10.1371/journal.pone.0082521 (PMC3858316; doi:10.1371/journal.pone.0082521)

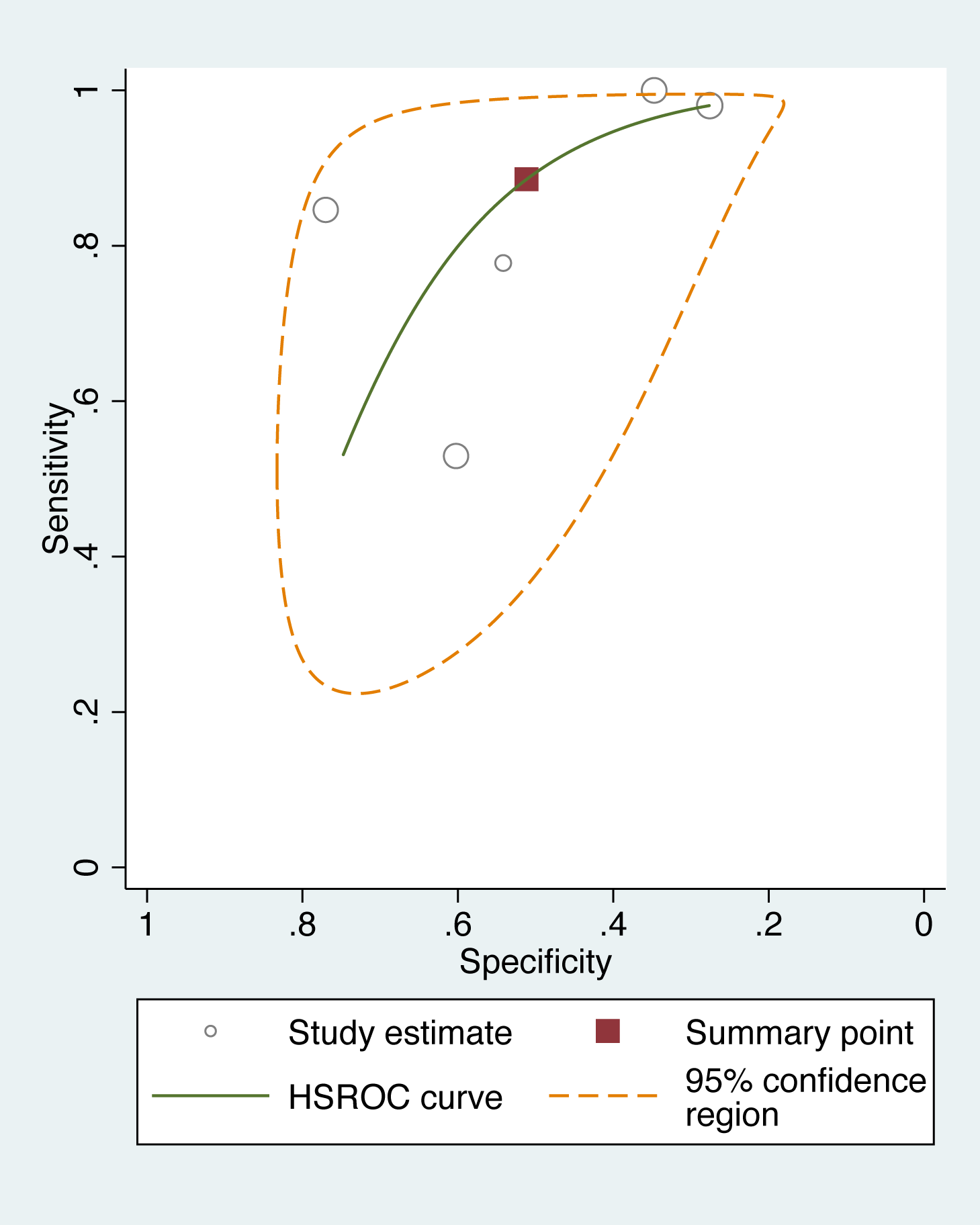

Supplement: Figure S1 — Summary ROC curve plot. The diagnosis of perinatal depression was based on the EPDS at the cut-off score ≥7, with pooled sensitivity and specificity calculated using bivariate meta-analysis. (TIF) [file pone.0082521.s002.tif]

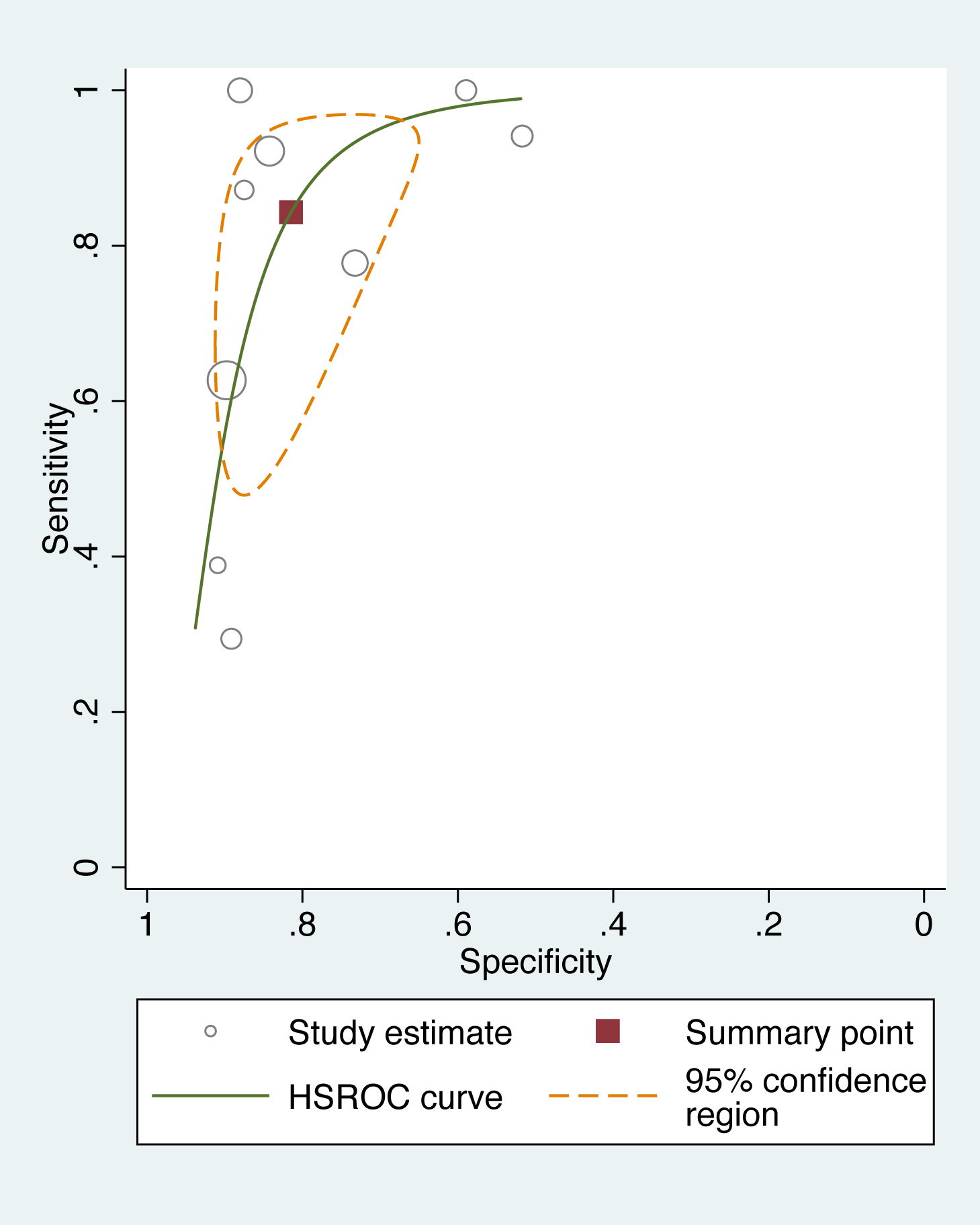

Supplement: Figure S2 — Summary ROC curve plot. The diagnosis of perinatal depression was based on the EPDS at the cut-off score ≥10, with pooled sensitivity and specificity calculated using bivariate meta-analysis. (TIF) [file pone.0082521.s003.tif]

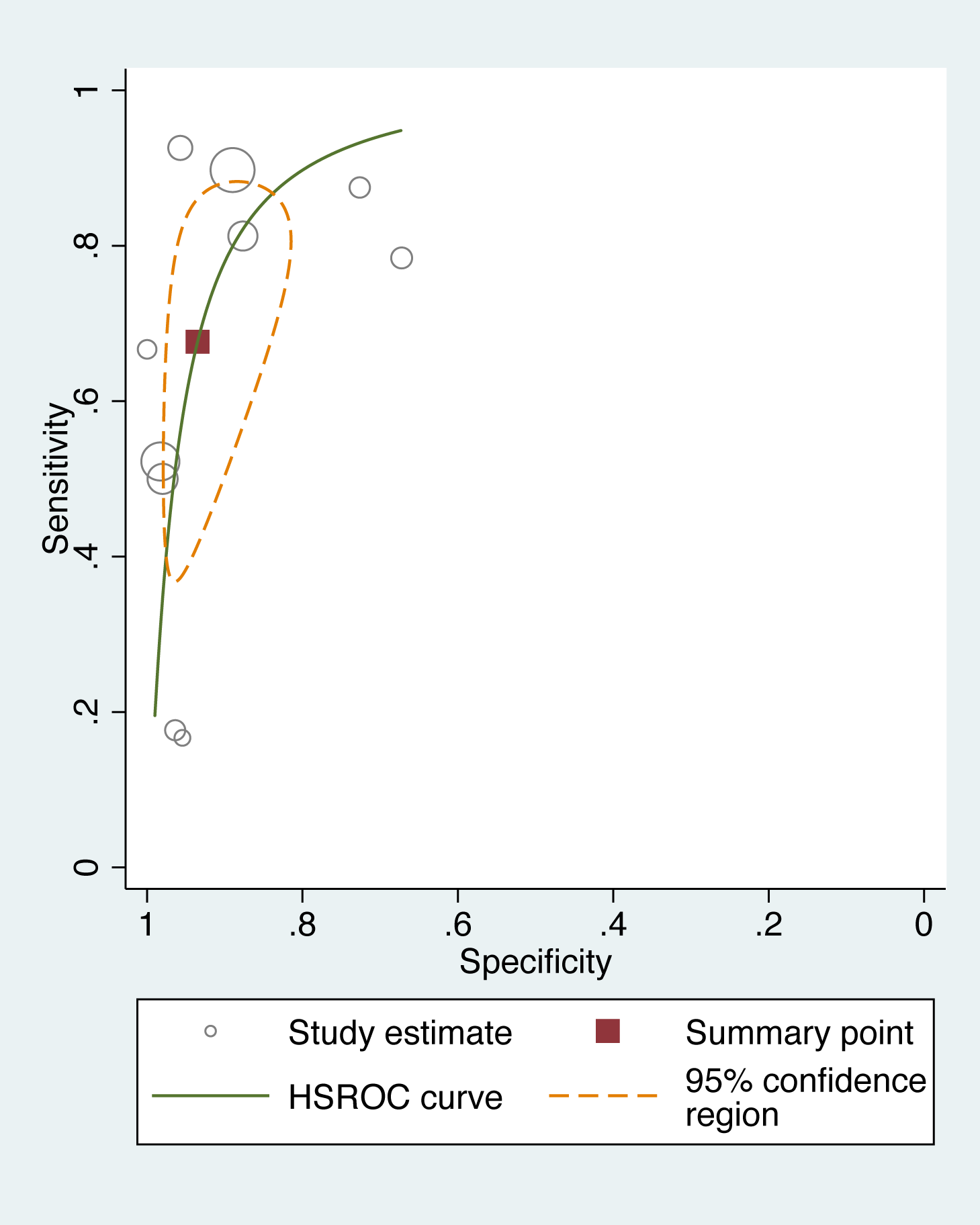

Supplement: Figure S3 — Summary ROC curve plot. The diagnosis of perinatal depression was based on the EPDS at the cut-off score ≥12, with pooled sensitivity and specificity calculated using bivariate meta-analysis. (TIF) [file pone.0082521.s004.tif]

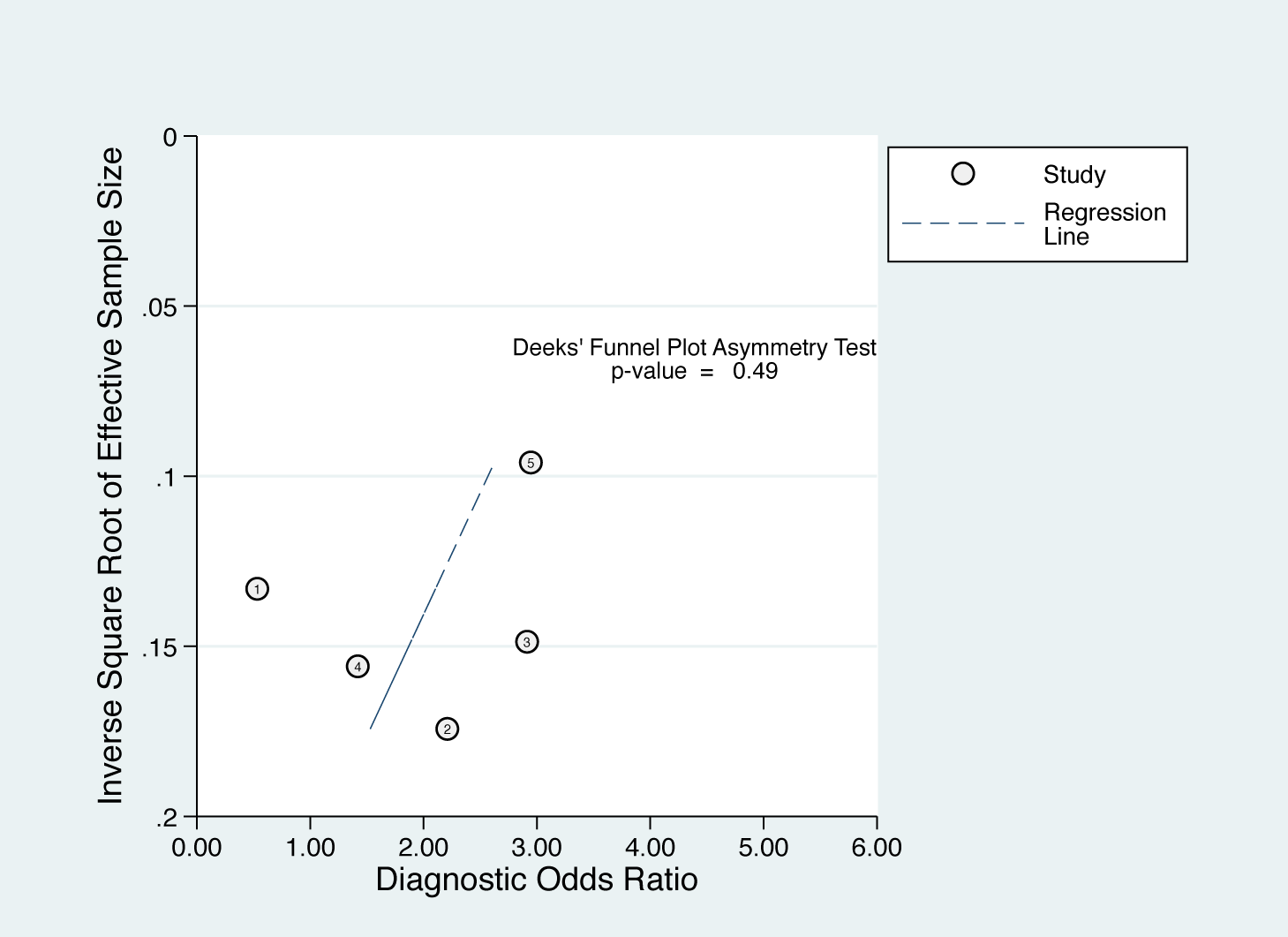

Supplement: Figure S4 — Asymmetry plot for EPDS at cut-off score ≥7. (TIF) [file pone.0082521.s005.tif]

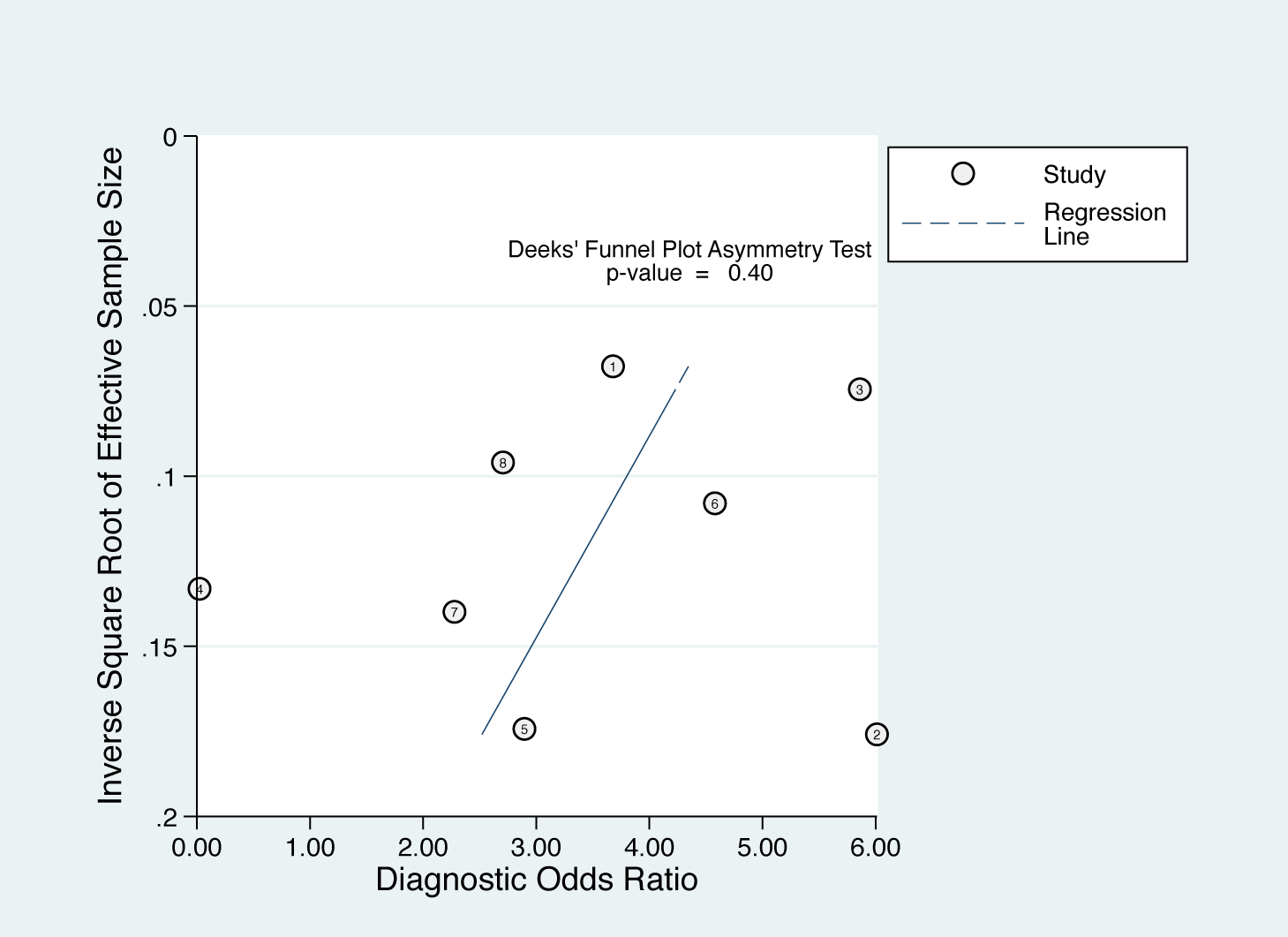

Supplement: Figure S5 — Asymmetry plot for EPDS at cut-off score ≥9. (TIF) [file pone.0082521.s006.tif]

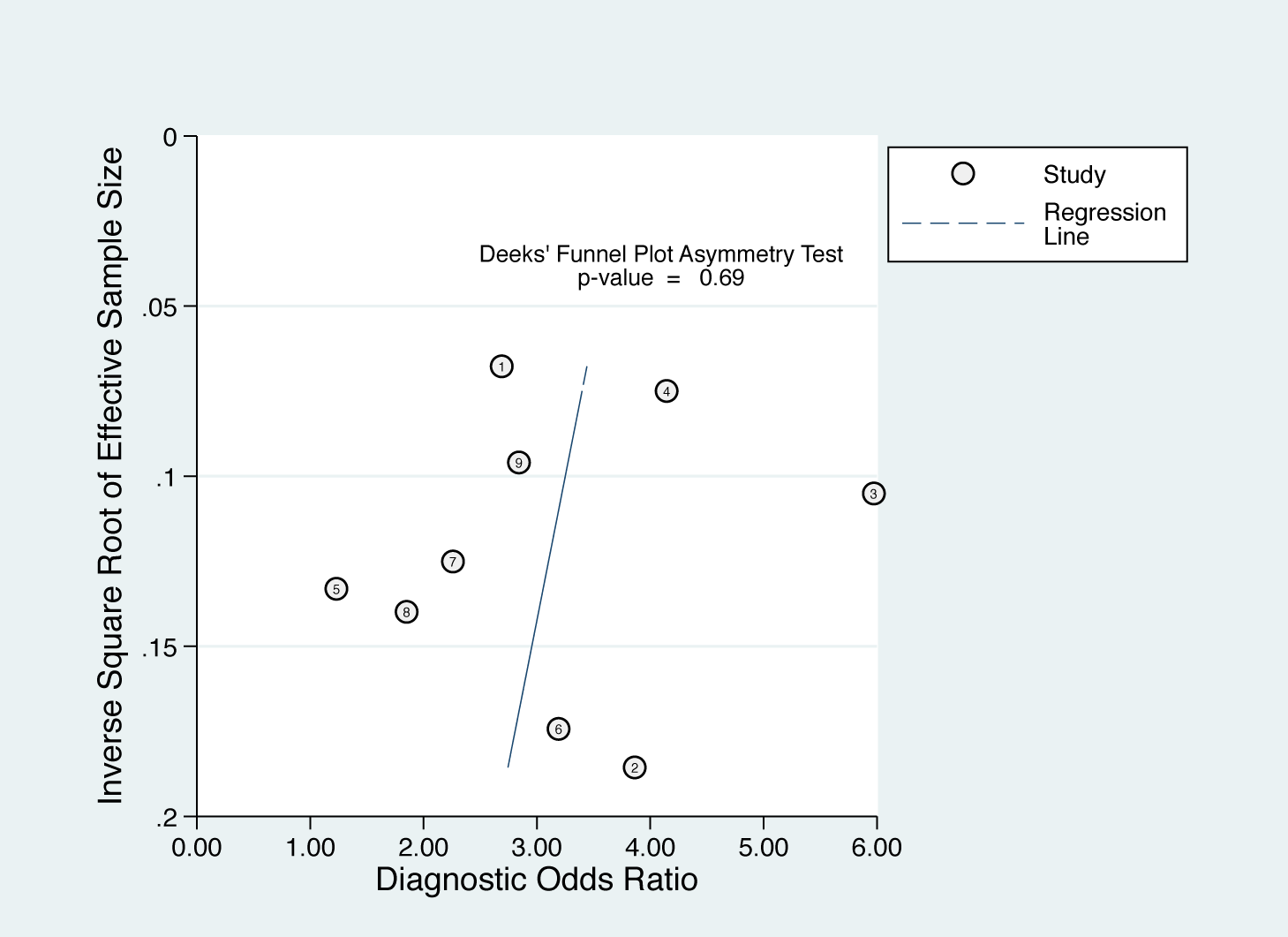

Supplement: Figure S6 — Asymmetry plot for EPDS at cut-off score ≥10. (TIF) [file pone.0082521.s007.tif]

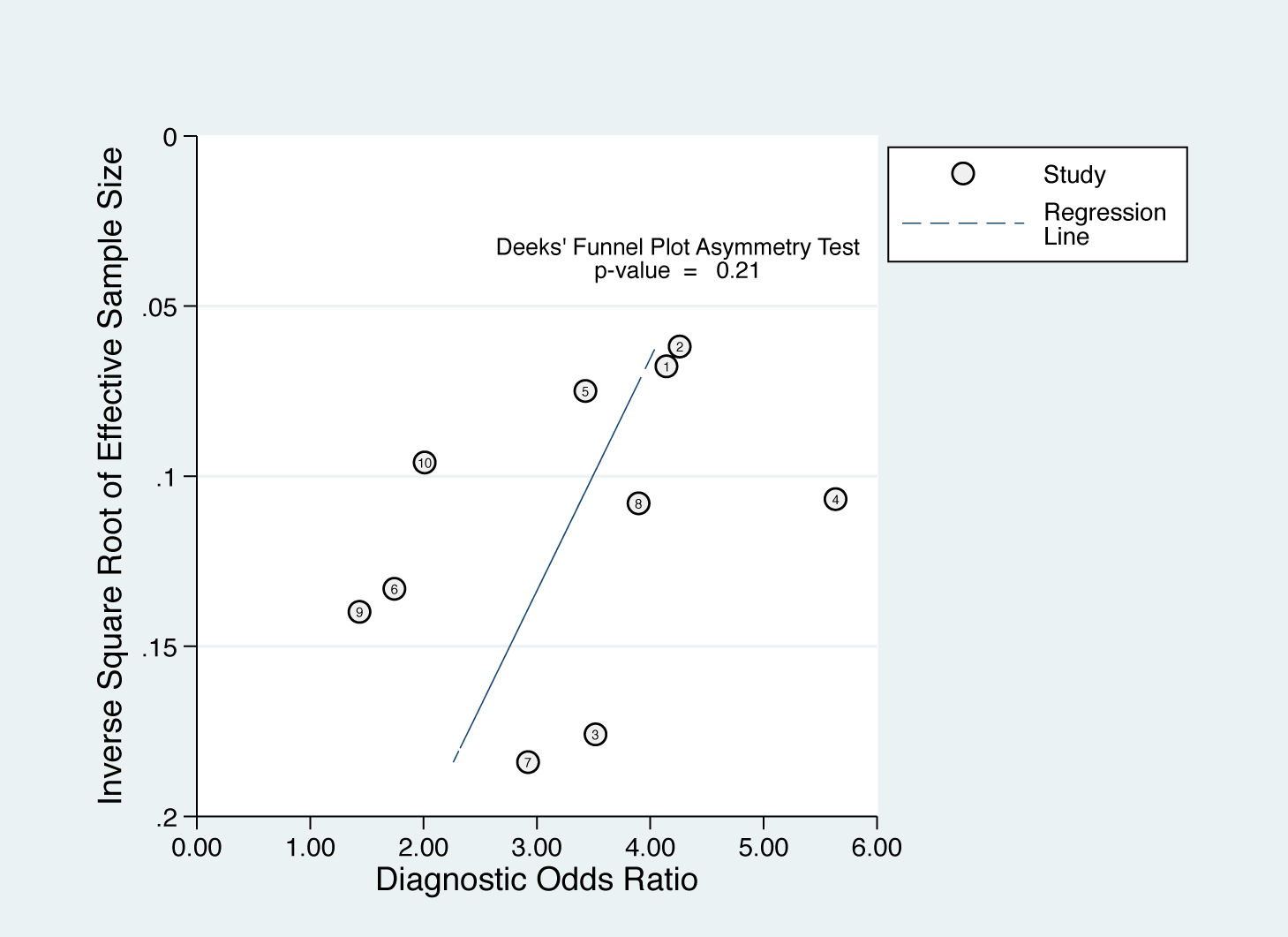

Supplement: Figure S7 — Asymmetry plot for EPDS at cut-off score ≥12. (TIF) [file pone.0082521.s008.tif]
