# Supplementary material for: Reliability and Validity of Instruments for Assessing Perinatal Depression in African Settings: Systematic Review and Meta-Analysis
Source: PLoS One. 2013 Dec 10;8(12):e82521. doi: 10.1371/journal.pone.0082521 (PMC3858316; doi:10.1371/journal.pone.0082521)
Supplement: Table S1 — Search terms applied to electronic databases. All database searches were completed on January 27, 2012, with the exception of searches conducted using the African Journal Archive, African Journals Online, and the World Health Organization African Index Medicus (which were completed May 30, 2012). The Medical Literature Analysis and Retrieval System Online search was updated on January 23, 2013. (PDF) [file pone.0082521.s009.pdf]

Table S1. Search terms applied to electronic databases. All database searches were completed on January 27, 2012, with the exception of searches conducted using the African Journal Archive, African Journals Online, and the World Health Organization African Index Medicus (which were completed May 30, 2012). The Medical Literature Analysis and Retrieval System Online search was updated on January 23, 2013.

Database	Search terms
The African Journal Archive	depression OR distress
African Journals Online	(depressive OR depression OR affective OR mood OR postpartum OR postnatal) AND (diagnosis OR sensitivity OR specificity OR validation OR screening OR psychometric OR "factor analysis" OR "factor structure" OR reliability OR validity OR consistency)
Cumulative Index to Nursing and Allied Health Literature	(MH “affective disorders” OR MH “Beck Depression Inventory, revised edition” OR MH “Center for Epidemiological Studies Depression scale” OR MH “Edinburgh Postnatal Depression Scale” OR MH “Geriatric Depression Scale” OR MH “Hamilton Rating Scale for Depression” OR MH “Self-Rating Depression Scale” OR MH “Bech-Rafaelson Melancholia Scale” OR AB depressive OR AB depression OR AB “psychological distress” OR AB idiom) AND (MH Africa OR MH refugees OR AB Africa) AND (MH diagnosis OR MH psychometrics OR MH “factor analysis” OR MH “reliability and validity” OR MH “rasch analysis” OR AB “construct validity” OR AB “convergent validity” OR AB “divergent validity” OR AB “discriminant validity” OR AB “content validity” OR AB “face validity” OR AB “criterion validity” OR AB “concurrent validity” OR AB “predictive validity” OR AB validity OR AB reliability OR AB consistency)
Embase	(depression:cl OR “distress syndrome”:cl OR “Bech-Rafaelsen Melancholia Scale”:cl OR “Beck Depression Inventory”:cl OR “Beck Hopelessness Scale”:cl OR “Brief Psychiatric Rating Scale”:cl OR “depression inventory”:cl OR “Diagnostic Interview Schedule”:cl OR “Edinburgh Postnatal Depression Scale”:cl OR “General Health Questionnaire”:cl OR “Geriatric Depression Scale”:cl OR “Hamilton scale”:cl OR “Montgomery Asberg Depression Rating Scale”:cl OR “Psychiatric Symptom Index”:cl OR “Self-rating Depression Scale”:cl OR “Symptom Checklist 90”:cl OR depressive:ab OR depression:ab OR “psychological distress”:ab OR idiom:ab) AND (Africa:cl OR refugee:cl OR Africa:ab) AND (“psychiatric diagnostic”:cl OR diagnosis:cl OR “sensitivity and specificity”:cl OR validity:cl OR reliability:cl OR “mass screening”:cl OR “factorial analysis”:cl OR “Rasch analysis”:cl OR psychometry:cl OR screening:ab OR psychometric:ab OR “factor analysis”:ab OR “factor structure”:ab OR “Rasch analysis”:ab OR “latent trait analysis”:ab OR “construct validity”:ab OR “convergent validity”:ab OR “divergent validity”:ab OR “discriminant validity”:ab OR “content validity”:ab OR “face validity”:ab OR “criterion validity”:ab OR “concurrent validity”:ab OR “predictive validity”:ab OR “validity”:ab OR “reliability”:ab OR “consistency”:ab)

Medical Literature Analysis and Retrieval System Online	(“depressive disorder”[MeSH Terms] OR “depression”[MeSH Terms] OR “affective symptoms”[MeSH Terms] OR “mood disorders”[MeSH Terms] OR “depression, postpartum”[MeSH Terms] OR “stress, psychological”[MeSH Terms] OR “depressive”[TIAB] OR “depression”[TIAB] OR “psychological distress”[TIAB] OR “idiom”[TIAB]) AND (“Africa”[MeSH Terms] OR “refugees”[MeSH Terms] OR “Africa”[TIAB]) AND (“diagnosis”[MeSH Terms] OR “sensitivity and specificity”[MeSH Terms] OR “reproducibility of results”[MeSH Terms] OR “validation studies as topic”[MeSH Terms] OR “validation studies”[Publication Type] OR “screening”[TIAB] OR “psychometric”[TIAB] OR “factor analysis”[TIAB] OR “factor structure”[TIAB] OR “Rasch analysis”[TIAB] OR “latent trait analysis”[TIAB] OR “construct validity”[TIAB] OR “convergent validity”[TIAB] OR “divergent validity”[TIAB] OR “discriminant validity”[TIAB] OR “content validity”[TIAB] OR “face validity”[TIAB] OR “criterion validity”[TIAB] OR “concurrent validity”[TIAB] OR “predictive validity”[TIAB] OR “validity”[TIAB] OR “reliability”[TIAB] OR “consistency”[TIAB])
PsycINFO	(DE “affective disorders” OR DE “stress” OR AB “depressive” OR AB “depression” OR AB “psychological distress” OR AB “idiom”) AND (DE “african cultural groups” OR DE “refugees” or AB “Africa”) AND (DE “test validity” OR DE “statistical validity” OR DE “factor analysis” OR DE “screening” OR DE “psychometrics” OR AB “psychometric” OR AB “factor analysis” OR AB “factor structure” OR AB “Rasch analysis” OR AB “latent trait analysis” OR AB “construct validity” OR AB “convergent validity” OR AB “divergent validity” OR AB “discriminant validity” OR AB “content validity” OR AB “face validity” OR AB “criterion validity” OR AB “concurrent validity” OR AB “predictive validity” OR AB “validity” OR AB “reliability” OR AB “consistency”)
World Health Organization African Index Medicus	depression OR distress
