# Supplementary material for: Reliability and Validity of Instruments for Assessing Perinatal Depression in African Settings: Systematic Review and Meta-Analysis
Source: PLoS One. 2013 Dec 10;8(12):e82521. doi: 10.1371/journal.pone.0082521 (PMC3858316; doi:10.1371/journal.pone.0082521)
Supplement: Table S2 — List of studies included in the review. (PDF) [file pone.0082521.s010.pdf]

Table S2. List of studies included in the review

Citation	Study Design	Instrument(s) Assessed	Summary of Evidence for Reliability or Validity
Abiodun and colleagues [1]	Consecutive sample of 240 women attending an antenatal clinic at a tertiary care facility in western Nigeria, dates of data collection not provided. The English or Yoruba versions of the GHQ-30 were self-administered by literate patients and administered to illiterate patients. A research psychiatrist blinded to the results of the GHQ-30 evaluated each patient and administered the PSE schedule to establish the reference criterion of psychiatric 'caseness'.	GHQ-30	GHQ-30 ≥5 had 0.80 sensitivity and 0.81 specificity for detecting psychiatric 'caseness' (most commonly anxiety states and neurotic depression).
Abiodun [2]	Consecutive sample of 748 women aged 16 years and older in a university teaching hospital (medical/surgical [275], gynecology [233], antenatal [240]) and 330 women from a community setting in western Nigeria. The English or Yoruba versions of the GHQ-12 and depression subscale of the HADS were self-administered by literate patients and administered to illiterate patients. A research psychiatrist blinded to the results of the GHQ-12 and HADS evaluated each patient and administered the PSE schedule to establish the reference criterion of psychiatric 'caseness'.	GHQ-12<br>HADS	HADS depression subscale ≥8 had 0.90-0.92 sensitivity and 0.87-0.91 specificity, while the GHQ-12 ≥3 had 0.83-0.91 sensitivity and 0.78-0.83 specificity, for detecting psychiatric 'caseness' (most commonly neurotic depression and anxiety states).
Abiodun [3]	Consecutive sample of 360 women aged 15 years and older attending one of 3 primary care facilities for a 6-week postnatal visit in western Nigeria, dates of data collection not provided. The English or Yoruba versions of the 10-item EPDS were self-administered by literate patients and administered to illiterate patients. A research psychiatrist blinded to the results of the EPDS evaluated each patient and administered the PSE schedule to establish the reference criterion diagnosis of postnatal depression.	EPDS	EPDS ≥9 had 0.88 sensitivity and 0.84 specificity for detecting postnatal depression.
Aderibigbe and colleagues [4]	Consecutive sample of 277 women attending a university hospital antenatal clinic in Nigeria self-administered the English version of the GHQ-28, or were administered the Yoruba version by a trained research assistant. A subsample of 106 participants, including each participant with a GHQ-28 ≥5, and the next participant with a GHQ-28 <5 interviewed after each participant with a GHQ-28 ≥5, was selected for an in-depth assessment using the Yoruba version of the PAS to establish the reference criterion of psychiatric 'caseness'.	GHQ-28	Factor analysis revealed a four-factor solution accounting for 40% of the variance: somatic symptoms, anxiety and insomnia, social dysfunction, and severe depression.

Aderibigbe and Gureje [5]	See above	GHQ-28	The GHQ-28 had a statistically significant correlation with the PAS score (Pearson's r=0.31, P<0.001). GHQ-28 ≥4 had 0.75 sensitivity and 0.83 specificity for detecting psychiatric 'caseness' (most commonly major depression, generalized anxiety disorder, and agoraphobia).
Adewuya and colleagues [6]	Consecutive sample of 876 women attending a six-week postnatal appointment at five health centres in the semi-urban town of Ilesa, Nigeria were recruited, along with 900 matched women from the general medical practice. Literate women self-administered English or Yoruba versions of the EPDS and 21-item BDI, while illiterate women were administered these scales by one of the study authors. Two psychiatrists blinded to the EPDS and BDI scores administered the SCID to all women with EPDS ≥9 or BDI ≥10 and a randomly selected sample of 875 women with EPDS <9 and BDI <10 to establish the DSM-III reference criterion diagnoses or major or minor depressive disorder.	EPDS<br>BDI	The EPDS had an internal consistency of 0.89. There was a statistically significant association between the EPDS and BDI scores (Spearman's rho=0.46, P<0.001). EPDS ≥9 had 0.94 sensitivity and 0.97 specificity for detecting major or minor depression (AUC=0.99), and 1.00 sensitivity and 0.89 specificity for detecting major depression only. BDI ≥10 had 0.89 sensitivity and 0.97 specificity for detecting major or minor depression (AUC=0.92).
Adewuya [7]	Consecutive sample of 478 women in the semi-urban town of Ilesa, Nigeria were recruited from five health centers on the day after delivery. At 5 days postnatally, women self-administered English or Yoruba versions of the MBS and the EPDS. At weeks 4 and 8 postnatally, two trained psychiatrists administered the SADS to establish the reference criterion diagnosis, and participants self-administered the EPDS.	MBS<br>EPDS	At 5 days postnatally, the MBS and EPDS scores had a statistically significant correlation (Spearman's rho=0.69, P<0.001). At 8 weeks postnatally, the EPDS ≥9 had 0.90 sensitivity and 0.89 specificity for detecting postnatal depression (AUC=0.94).
Adewuya and colleagues [8]	Consecutive sample of 182 women in the semi-urban town of Ilesa, Nigeria were recruited from five health centers in late pregnancy (greater than 32 weeks' gestation). Literate women self-administered English or Yoruba versions of the EPDS, while illiterate women were administered the EPDS by a trained research assistant. All women with EPDS ≥6, and a random subset of 11 women with EPDS <6, underwent a MINI diagnostic assessment by a psychiatrist blinded to the EPDS scores to establish the reference criteria of major and minor depressive disorder.	EPDS	The EPDS had an internal consistency of 0.85. EPDS ≥12 had 1.00 sensitivity and 0.96 specificity for detecting major depression (AUC=0.995). EPDS ≥10 had 0.87 sensitivity and 0.92 specificity for detecting major or minor depression (AUC=0.97).

Agoub and colleagues [9]	Convenience sample of 144 women in Morocco who had given birth in the two months prior. Participants were recruited at first postnatal visit 15-20 days after delivery and reassessed at 6 weeks, 6 months, and 9 months. The Arabic version of the EPDS was self-administered, verbally administered for illiterate participants. The Moroccan Colloquial Arabic version of the MINI was used to establish the reference criterion diagnosis of major depressive disorder.	EPDS	EPDS ≥12 had 0.92 sensitivity and 0.96 specificity for detecting major depressive disorder.
Baggaley and colleagues [10]	61 women were administered West African French and local-language (Moore and Dioula) versions of the K10/K6 at 3 and 6 months postnatally. A local psychiatrist blinded to the K10/K6 score conducted diagnostic interviews to establish the reference criterion diagnosis of depression consistent with ICD-10 criteria.	K10<br>K6	The internal consistency of the K10 was 0.87, and the internal consistency of the K6 was 0.78. K10 ≥12 had 0.74 sensitivity and 0.76 specificity for detecting clinically diagnosed depression (AUC=0.77). K6 ≥10 had 0.59 sensitivity and 0.85 specificity (AUC=0.75) for detecting clinically diagnosed depression.
Bass and colleagues [11]	A convenience sample of 80 women in Kinshasa, DRC who had given birth to a child within the previous two years was asked to free-list problems faced by new mothers in the first postnatal year. A convenience sample of 14 key informants (traditional healers, ministers, marriage counselors, local older women) were interviewed in-depth about postnatal mental health problems. The qualitative data were used to develop a new screening instrument based on the EPDS and HSCL-15; two poorly understood items were dropped (from the EPDS) and seven non-overlapping items identified as being salient symptoms of a local syndrome (*malady ya souci*) were added. A purposive sample of 133 women who had recently given birth to a child was administered the 23-item total symptom scale. For the reference criterion, 'caseness' was established if there was agreement between the participant and key informant about whether she had *malady ya souci*. A random sample of women were re-interviewed within 3 days of the first assessment.	HSCL<br>EPDS-8<br>14-item locally derived scale	Internal consistency was 0.86 for the HSCL, 0.76 for the EPDS-8, 0.88 for the 14-item locally derived instrument, and 0.92 for the 23-item total symptom scale. Correlations between the first and second assessments were 0.59 for the HSCL (P<0.001), 0.53 for the EPDS-8 (P=0.003), and 0.42 for the 23-item total symptom scale (P=0.02). The 14-item locally derived instrument had a statistically significant association with a locally derived functional impairment scale (P<0.001). With regards to the reference criterion of psychiatric caseness, HSCL ≥15 had 0.81 sensitivity and 0.80 specificity (AUC=0.87), EPDS-8 ≥8 had 0.85 sensitivity and 0.75 specificity (AUC=0.83), the 14-item locally derived instrument at a cutoff of ≥16 had 0.81 sensitivity and 0.80 specificity (AUC=0.85), and the 23-item total symptom scale score ≥25 had 0.81 sensitivity and 0.85 specificity (AUC=0.87).
De Bruin and colleagues [12]	A sample of all 147 women living in a peri-urban settlement near Cape Town, South Africa who had recently given birth were administered the isiXhosa version of the EPDS.	EPDS	The EPDS had an internal consistency of 0.89. Maximum likelihood confirmatory factor analysis was used to test two measurement models: (a) a one-dimensional construct, and (b) two correlated factors of depressive feelings and cognitive anxiety. The root mean square error of approximation values indicated that both the one- and two-factor models provided a satisfactory fit to the data and that the two-factor model only provided a marginally improved fit.

Chibanda and colleauges [13]	A random sample of 210 women attending two primary care clinics in peri-urban Zimbabwe were interviewed at 6 weeks postnatally. Trained community counselors administered a Shona version of the EPDS. Two psychiatrists blinded to the EPDS scores assessed the women using DSM-IV criteria to establish the reference criterion diagnosis of major depression.	EPDS	The internal consistency of the EPDS was 0.87. EPDS ≥12 had 0.88 sensitivity and 0.89 specificity for detecting major depression (AUC=0.82).
Hanlon and colleauges [14]	A community-based sample of 1285 consecutive women in rural Ethiopia were interviewed during the perinatal period and administered the Amharic 10-item EPDS, 20-item SRQ, and 29-item culturally modified SRQ-F across several studies. The CPRS was administered by psychiatry trainees to establish the reference criterion of clinically significant psychiatric morbidity.	EPDS<br>SRQ<br>SRQ-F	Two EPDS items did not translate well. Participants had difficulty understanding the four response options, so these were elicited using a two-stage process. Standardized examples were attached to three of the items that were not well understood. The EPDS had an internal consistency of 0.47. EPDS ≥6 had 0.77 sensitivity and 0.36 specificity for detecting common mental disorders (most commonly major depressive disorder and generalized anxiety disorder), with AUC=0.56. In the first validation study, the SRQ had an internal consistency of 0.84. SRQ ≥3 had 0.86 sensitivity and 0.86 specificity for detecting clinically significant psychiatric morbidity (AUC=0.82). In the second validation study, the SRQ had an internal consistency of 0.88, and SRQ ≥7 had 0.68 sensitivity and 0.62 specificity (AUC=0.70). SRQ scores had statistically significant associations with hunger (P<0.001), indebtedness (P<0.001), poor marital relationship quality (P<0.001), low perceived social support (P<0.001), stressful life events (P<0.001), violence during pregnancy (P<0.001), and poor health (P<0.001).
Hartley and colleagues [15]	All pregnant women (18+ years) in 24 neighborhoods of a peri-urban settlement near Cape Town, South Africa were recruited for participation in a randomized trial of family health. They were administered the isiXhosa version of the EPDS.	EPDS	The EPDS had an internal consistency of 0.87. EPDS ≥14 was associated with single motherhood, being unemployed, low income, alcohol use, experience of intimate partner violence, and poor financial and/or social support (all P<0.05).

Kaaya and colleagues [16]	A sample of 903 HIV-positive pregnant women enrolled in a randomized controlled trial in urban Tanzania were administered the 25-item HSCL. Psychiatrists blinded to the HSCL scores evaluated a subset of 100 participants using the SCID to establish the reference criterion diagnosis of major depressive disorder.	HSCL-15<br>HSCL-8	Internal consistency was 0.90 for the depression subscale (HSCL-15) and 0.93 overall (HSCL-25). Factor analysis performed on all 25 items revealed a single factor representing symptoms of depression and anxiety that explained 39% of the variance. HSCL-25 ≥1.06 had 0.89 sensitivity and 0.80 specificity for detecting major depressive disorder (AUC=0.86). HSCL-15 ≥1.03 had 0.89 sensitivity and 0.79 specificity (AUC=0.86). The HSCL-25 and HSCL-15 both had statistically significant associations with perceived social support ($P<0.01$) and all 8 dimensions of the Medical Outcomes Study Short Form 36-Item Health Survey (SF-36) (each $P<0.01$). Eight items selected after ROC analysis were used to create a modified depression subscale (HSCL-8) which, at a cutoff of ≥1.06, had 0.89 sensitivity and 0.85 specificity for detecting major depressive disorder (AUC=0.88).
Kaaya and colleagues [17]	In-depth interviews were conducted with 10 key informants, including women's group and village leaders, traditional healers, village health workers, and with 10 women identified by key informants as experiencing symptoms of locally defined syndromes. These interviews produced 30 different local idioms of depressive and anxiety symptoms, which were administered in Kiswahili along with 17 of the semantically and conceptually distinct items from the HSCL to a convenience sample of 787 pregnant women attending an antenatal clinic in Tanzania at 28-36 weeks gestation. Intra-class correlation coefficients were used to estimate both inter-rater reliability (using a subsample of 21 participants) and test-retest reliability (using a subsample of 12 participants interviewed 1 week after the initial interview).	DSQ-19	Stepwise forward logistic regression was used to select 19 of the 47 items for inclusion in the new scale, the DSQ-19. The DSQ-19 had an internal consistency of 0.84. The intra-class correlation between interviewers was 0.89, and the intra-class correlation within participants was 0.82. Principal components analysis revealed a single factor. DSQ-19 scores had statistically significant correlations with the SF-36, satisfaction with economic wellbeing, household decision-making power, and marital status (P-values not reported).
Kaaya and colleagues [18]	Qualitative interviews were conducted in Kiswahili with a purposive sample of 12 traditional practitioners and 10 women previously affected by depression living in a peri-urban region near Dar es Salaam, Tanzania. Axial coding was used to identify and describe clusters of idioms of distress.		One categorization of pregnancy that emerged from the interviews, the "problematic pregnancy," framed women's recollection of distress. The data suggested existence of a construct with similarities to Western-based psychological understandings of depression.
Lawrie and colleauges [19]	A consecutive sample of 103 women attending a 6-week postnatal clinic at a hospital in urban South Africa were administered the EPDS in one of six South African languages. A physician blinded to the EPDS scores conducted structured psychiatric interviews guided by DSM-IV criteria and the MADRS to establish the reference criterion diagnoses of major and minor depression.	EPDS	EPDS ≥12 had 1.00 sensitivity and 0.68 specificity for detecting major depression, and 0.80 sensitivity and 0.77 specificity for detecting major and minor depression combined.

Lee and colleagues [20]	The 25-item HSCL was administered in Kiswahili to a convenience sample of 787 pregnant women attending an antenatal clinic in Tanzania at 28-36 weeks gestation. Intra-class correlation coefficients were used to estimate both inter-rater reliability (using a subsample of 21 participants) and test-retest reliability (using a subsample of 12 participants interviewed 1 week after the initial interview).	HSCL-15	Internal consistency was 0.90 for the HSCL-25 and 0.88 for the 15-item depression subscale. The intra-class correlation between interviewers was 0.85, and the intra-class correlation within participants was 0.85. Principal components analysis revealed a single factor. The HSCL-25 and HSCL-15 had statistically significant correlations with 4 dimensions of the SF-36 (P-values not reported).
Nhiwatiwa and colleauges [21]	A consecutive sample of 500 pregnant women seen at antenatal clinics or by traditional birth attendants in the community were enrolled at 32 weeks' gestation into a prospective cohort study in peri-urban Zimbabwe. The 14-item SSQ was administered to all participants. For all 95 women with SSQ ≥8, and for a random sample of 105 women with SSQ <8, the Shona version of the CISR was administered by a psychiatrist at 6-8 weeks postnatally to establish the reference criterion of psychiatric 'caseness'.	SSQ	SSQ had statistically significant associations with poor intimate partner relationship quality and worsened health status. SSQ ≥8 at 32 weeks' gestation had 0.82 sensitivity and 0.66 specificity for detecting psychiatric 'caseness' (most commonly postnatal depression).
Rochat [22]	This study recruited a consecutive sample of 112 women (16+ years) attending a first (for the current pregnancy) antenatal visit and testing for HIV as part of a PMTCT program in rural KwaZulu-Natal, South Africa. They were administered the EPDS, along with the SCID to establish the reference criterion diagnosis of major depressive episode. A subset of 55 consecutive women (27 HIV-positive and 28 HIV-negative) were recruited to participate in a qualitative sub-study. Zulu-speaking research assistants, clinical psychologists, and a psychiatric nurse participated in a one-day translation workshop to guide translation of the EPDS and SCID into Zulu.	EPDS	Focus group participants identified the IsiZulu word *ingcindezi* ("for something to be pressing down on you or weighing down on you and your emotions") as a word commonly used in this population to describe depression and its social and psychological sequelae. The EPDS had an internal consistency of 0.61. EPDS ≥13 had 0.69 sensitivity and 0.78 specificity for detecting major depression (AUC=0.73). In the qualitative study, interpersonal conflict, unwanted pregnancy, and testing positive for HIV were prominent themes leading to emotional distress.
Spies and colleagues [23]	The sample of 129 women with low-risk pregnancies at less than 20 weeks' gestation presenting for a first antenatal visit at midwife obstetric units in peri-urban South Africa was drawn from a larger prospective cohort study. The K10 was self-administered in English or Afrikaans. A researcher administered the SCID to establish the reference criterion diagnosis of current major depressive episode.	K10	K10 ≥21.5 had 0.73 sensitivity and 0.54 specificity for detecting a current major depressive episode (AUC=0.66).
Taiwo and Olayinka [24]	A convenience sample of 256 women who gave birth at a teaching hospital in north central Nigeria self-administered the English or Hausa version of the EPDS at 6 weeks postnatally. All 24 women with EPDS ≥12, and a random sample of 38 women with EPDS <12, underwent a clinical diagnostic interview by a psychiatrist to establish the reference criterion diagnosis of major depression.	EPDS	EPDS ≥7 had 0.72 sensitivity and 0.62 specificity for detecting major depression.

Tesfaye and colleagues [25]	The EPDS and K6/K10 were administered to a pilot sample of 30 postnatal women attending vaccination clinics at a primary health care center in Addis Ababa, Ethiopia, and they were further probed to explore potentially unclear items. A convenience sample of 100 postnatal women (18-38 years) attending vaccination clinics at two primary health care centers in Ethiopia were administered the EPDS and K6/K10. Psychiatry residents blinded to the EPDS and K6/K10 scores conducted clinical interviews, guided by the CPRS and DSM-IV, to establish the reference criterion diagnosis of major or minor depressive disorder.	EPDS<br>K6<br>K10	The authors describe several scale items that were not well understood by participants in the pilot sample. The internal consistency coefficients were 0.71 for the EPDS (and 0.79 when two problematic items were removed from analysis), 0.86 for the K6, and 0.90 for the K10. In the detection of major or minor depression (combined), EPDS ≥8 had 0.85 sensitivity and 0.77 specificity (AUC=0.86), K6 ≥6 had 0.85 sensitivity and 0.81 specificity (AUC=0.91), and K10 ≥8 had 0.85 sensitivity and 0.76 specificity (AUC=0.91).
Uwakwe and Okonkwo [26]	A consecutive sample of 225 women (18-29 years) who were either in the maternity ward of a peri-urban Nigerian teaching hospital on postnatal day 7 or attending its postnatal clinic self-administered the English or Igbo versions of the EPDS and 20-item ZDS. Illiterate patients were administered the scales by resident physicians. A psychiatrist conducted a clinical diagnostic interview, guided by a modified version of the ICD-10 Symptom Checklist, to establish the reference criterion diagnosis of depression.	EPDS<br>ZDS	Internal consistency was 0.83 for the EPDS and 0.79 for the ZDS. EPDS ≥9 had 0.75 sensitivity and 0.97 specificity for detecting depression.
Weobong and colleagues [27]	Among women (15-45 years) participating in an ongoing randomized, controlled trial, those from a single district in rural Ghana were interviewed at 5-11 weeks postnatally using the SRQ, EPDS, and PHQ-9. A clinical psychologist blinded to the results of the SRQ, EPDS, and PHQ-9 conducted clinical assessments using the CPRS to establish the reference criterion of psychiatric ‘caseness’. The intra-class correlation coefficient was used to estimate test-retest reliability in a subset of 40 women who were re-interviewed 2 weeks after the initial interview.	SRQ<br>EPDS<br>PHQ-9	Internal consistency was 0.79 for the EPDS, 0.78 for the SRQ, and 0.79 for the PHQ-9. Intra-class correlation coefficients were 0.51 for the EPDS and 0.75 for the PHQ-9. In relation to the reference criterion of psychiatric caseness, EPDS ≥11 had 0.78 sensitivity and 0.73 specificity (AUC=0.84), SRQ ≥7 had 0.77 sensitivity and 0.63 specificity (AUC=0.74), and PHQ-9 ≥5 had 0.94 sensitivity and 0.75 specificity (AUC=0.90).

AUC = area under the receiver-operating characteristic curve; BDI = Beck Depression Inventory; CISR = Revised Clinical Interview Schedule; CPRS = Comprehensive Psychopathological Rating Scale; DSQ = Dar-es-Salaam Symptom Questionnaire; EPDS = Edinburgh Postnatal Depression Scale; GHQ = General Health Questionnaire; HADS = Hospital Anxiety and Depression Scale; MADRS = Montgomery-Asberg Depression Rating Scale; MBS = Maternity Blues Scale; MINI = Mini International Neuropsychiatric Interview; PAS = Psychiatric Assessment Schedule; PMTCT = prevention of mother-to-child transmission; PSE = Present State Examination; SADS = Schedule for Affective Disorders and Schizophrenia; SCID = Structured Clinical Interview for the Diagnostic and Statistical Manual of Mental Disorders; SRQ = Self-Reporting Questionnaire; SSQ = Shona Symptom Questionnaire; ZDS = Zung Self-Rating Depression Scale

References (Table S2)

1. Abiodun OA, Adetoro OO, Ogunbode OO (1993) Psychiatric morbidity in a pregnant population in Nigeria. Gen Hosp Psychiatry 15: 125-128.

2. Abiodun OA (1994) A validity study of the Hospital Anxiety and Depression Scale in general hospital units and a community sample in Nigeria. Br J Psychiatry 165: 669-672.

3. Abiodun OA (2006) Postnatal depression in primary care populations in Nigeria. Gen Hosp Psychiatry 28: 133-136.

4. Aderibigbe YA, Riley W, Lewin T, Gureje O. (1996) Factor structure of the 28-item General Health Questionnaire in a sample of antenatal women. Int J Psych Med 26: 263-269.

5. Aderibigbe YA, Gureje O (1992) The validity of the 28-item General Health Questionnaire in a Nigerian antenatal clinic. Soc Psychiatry Psychiatr Epidemiol 27: 280-283.

6. Adewuya AO, Eegunranti AB, Lawal AM (2005) Prevalence of postnatal depression in Western Nigerian women: a controlled study. Int J Psychiatr Clin Pract 9: 60-64.

7. Adewuya AO (2006) Early postpartum mood as a risk factor for postnatal depression in Nigerian women. Am J Psychiatry 163: 1435-1437.

8. Adewuya AO, Ola BA, Dada AO, Fasoto OO (2006) Validation of the Edinburgh Postnatal Depression Scale as a screening tool for depression in late pregnancy among Nigerian women. J Psychosom Obstet Gynaecol 27: 267-272.

9. Agoub M, Moussaoui D, Battas O (2005) Prevalence of postpartum depression in a Moroccan sample. Arch Womens Ment Health 8: 37-43.

10. Baggaley RF, Ganaba R, Filippi V, Kere M, Marshall T, et al. (2007) Detecting depression after pregnancy: the validity of the K10 and K6 in Burkina Faso. Trop Med Int Health 12: 1225-1229.

11. Bass JK, Ryder RW, Lammers MC, Mukaba TN, Bolton PA (2008) Post-partum depression in Kinshasa, Democratic Republic of Congo: validation of a concept using a mixed-methods cross-cultural approach. Trop Med Int Health 13: 1534-1542.

12. de Bruin GP, Swartz L, Tomlinson M, Cooper PJ, Molteno C. (2004) The factor structure of the Edinburgh Postnatal Depression scale in a South African peri-urban settlement. S Afr J Psychol 34: 113-121.

13. Chibanda D, Mangezi W, Tshimanga M, Woelk G, Rusakaniko P, et al. (2010) Validation of the Edinburgh Postnatal Depression Scale among women in a high HIV prevalence area in urban Zimbabwe. Arch Womens Ment Health 13: 201-206.

14. Hanlon C, Medhin G, Alem A, Araya M, Abdulahi A, et al. (2008) Detecting perinatal common mental disorders in Ethiopia: validation of the self-reporting questionnaire and Edinburgh Postnatal Depression Scale. J Affect Disord 108: 251-262.

15. Hartley M, Tomlinson M, Greco E, Comulada WS, Stewart J, et al. (2011) Depressed mood in pregnancy: prevalence and correlates in two Cape Town peri-urban settlements. Reproductive Health 8:9.

16. Kaaya SF, Fawzi MC, Mbwambo JK, Lee B, Msamanga GI, et al. (2002) Validity of the Hopkins Symptom Checklist-25 amongst HIV-positive pregnant women in Tanzania. Acta Psychiatr Scand 106: 9-19.

17. Kaaya SF, Lee B, Mbwambo JK, Smith-Fawzi MC, Leshabari MT. (2008) Detecting depressive disorder with a 19-item local instrument in Tanzania. Int J Soc Psychiatry 2008 54:21-33.

18. Kaaya SF, Mbwambo JK, Smith Fawzi MC, van den Borne H, Schaalma H, et al. (2010) Understanding women's experiences of distress during pregnancy in Dar es Salaam, Tanzania. Tanz J Health Res 12:1-12.

19. Lawrie TA, Hofmeyr GJ, de Jager M, Berk M (1998) Validation of the Edinburgh Postnatal Depression Scale on a cohort of South African women. S Afr Med J 88: 1340-1344.

20. Lee B, Kaaya SF, Mbwambo JK, Smith-Fawzi MC, Leshabari MT. (2008) Detecting depressive disorder with the Hopkins Symptom Checklist-25 in Tanzania. Int J Soc Psychiatry 54:7-20.

21. Nhiwatiwa S, Patel V, Acuda W (1998) Predicting postnatal mental disorder with a screening questionnaire: a prospective cohort study from Zimbabwe. J Epidemiol Community Health 52: 262-266.

22. Rochat TJ (2011) Depression among pregnant women testing for HIV in rural South Africa (Ph.D. dissertation). Stellenbosch: Stellenbosch University.

23. Spies G, Stein DJ, Roos A, Faure SC, Mostert J, et al. (2009) Validity of the Kessler 10 (K-10) in detecting DSM-IV defined mood and anxiety disorders among pregnant women. Arch Womens Ment Health 12: 69-74.

24. Taiwo OJ, Olayinka OO (2007) The validation of Edinburgh Postpartum Depression Scale (EPDS) in north central Nigeria. J Med Trop 9: 29-40.

25. Tesfaye M, Hanlon C, Wondimagegn D, Alem A (2010) Detecting postnatal common mental disorders in Addis Ababa, Ethiopia: validation of the Edinburgh Postnatal Depression Scale and Kessler Scales. J Affect Disord 122: 102-108.

26. Uwakwe R, Okonkwo JE (2003) Affective (depressive) morbidity in puerperal Nigerian women: validation of the Edinburgh Postnatal Depression Scale. Acta Psychiatr Scand 107: 251-259.

27. Weobong B, Akpalu B, Doku V, Owusu-Agyei S, Hurt L, et al. (2009) The comparative validity of screening scales for postnatal common mental disorder in Kintampo, Ghana. J Affect Disord 113: 109-117.
